# Comparative Analysis of Dietary Patterns in Children With Phenylketonuria Phenotypes and Controls: Implications for Nutritional Status

**DOI:** 10.1002/jimd.70196

**Published:** 2026-05-03

**Authors:** Dolores Garcia‐Arenas, Aida Ormazabal, Paula Isern, Blanca Barrau‐Martinez, Arnau Gonzalez‐Rodriguez, Alba Tor‐Roca, Rafael Llorach, Jaume Campistol‐Plana, Mireia Urpi‐Sarda, Consortium PKU.cat.

**Affiliations:** ^1^ Nutrition, Food Science and Gastronomy Department, Faculty of Pharmacy and Food Science Food Science and Nutrition Torribera Campus, University of Barcelona Barcelona Spain; ^2^ Inborn Errors of Metabolism Unit, Sant Joan de Déu Hospital Barcelona Spain; ^3^ Centro de Investigación Biomédica en Red de Enfermedades Raras (CIBERER), Instituto de Salud Carlos III Madrid Spain; ^4^ Clinical Biochemistry Department Sant Joan de Déu Hospital Barcelona Spain; ^5^ Institut de Recerca Sant Joan de Déu Barcelona Spain; ^6^ Institute for Research on Nutrition and Food Safety (INSA‐UB), Universitat de Barcelona Barcelona Spain; ^7^ Centro de Investigación Biomédica en Red Fragilidad y Envejecimiento Saludable (CIBERFES), Instituto de Salud Carlos III Madrid Spain; ^8^ Metabolic Unit, Neuropaediatrics Department Sant Joan de Déu Hospital Barcelona Spain

**Keywords:** children, insulin resistance, low natural protein diet, phenylalanine, phenylketonuria, physical activity

## Abstract

Individuals with phenylketonuria (PKU), caused by different variants of the phenylalanine hydroxylase gene, need to restrict their intake of phenylalanine. This study evaluated dietary patterns and physical activity levels in children with different PKU phenotypes compared to healthy controls. Eighty‐two children were recruited (22 classic PKU [cPKU], 21 BH_4_‐responsive PKU, 19 hyperphenylalaninemia, and 20 controls). Anthropometric data, dietary intake, biochemical markers, and physical activity were assessed. Classic PKU (cPKU) subjects exhibited higher carbohydrate and sugar intake than other PKU phenotypes and controls. Notably, 42% of carbohydrate and 17% of sugar intake was from special low‐protein foods, and 20% of carbohydrate and 29% of sugar intake was from protein substitutes. Compared to controls, the cPKU group was less physically active and reported a higher frequency of sweet consumption. Ninety percent of PKU had good metabolic control and carbohydrate intake was significantly correlated with HOMA‐IR; however, after adjusting for age, only a trend remained (*p* = 0.08). Participants in the PKU group following a low natural protein diet consumed more carbohydrate and sugars than those on a normal‐protein diet. Multivariate regression analysis showed that the low natural protein diet group was significantly associated with higher levels of vitamin B_12_, linoleic acid, α‐linolenic acid, eicosapentaenoic acid, and docosahexaenoic acid, and with lower levels of total cholesterol and HDL‐C compared to the normal‐protein diet group. In conclusion, children with PKU, particularly those with classical PKU following low‐protein diets, showed higher carbohydrate intake and distinct micronutrient and lipid profiles compared with healthy controls.

## 1 Introduction

Phenylketonuria (PKU) [OMIM: 261600] is an autosomal recessive disorder caused by a deficiency in phenylalanine hydroxylase, resulting in the inability to convert phenylalanine into tyrosine [1] Without appropriate treatment, PKU leads to severe intellectual disability, developmental delays, seizures, hypopigmentation, motor dysfunction, ataxia, and growth failure [1, 2].

Early diagnosis and adherence to a phenylalanine‐restricted diet enable most individuals with PKU to lead near‐normal lives. Additional therapeutic options, such as tetrahydrobiopterin (BH_4_), sepiapterin, and enzyme therapy with phenylalanine ammonia lyase, have further expanded the treatment landscape [3, 4, 5].

Dietary phenylalanine tolerance varies by phenylalanine hydroxylase gene variants and disease severity, ranging from mild to severe [6], and is influenced by growth or catabolism stage [7]. To meet nutritional needs, patients rely on protein substitutes (PS) consisting of phenylalanine‐free amino acids, glycomacropeptide, or large neutral amino acids, specialized low‐protein foods (SLPFs), and low protein foods such as fruits, vegetables, and fats. PS are a cornerstone of PKU management, supplying essential amino acids (except phenylalanine) and critical nutrients [7]. SLPFs are processed foods with reduced protein content typically high in carbohydrates and fats, differing substantially from conventional foods [8, 9, 10]. In severe PKU, both PS and SLPFs are vital for metabolic control their nutritional composition differs in calories, fats, carbohydrates, and sugars [10, 11], Okano et al. [12] reported a protein, fat, and carbohydrate ratio of 9.5:23.9:66.6%, indicating excess carbohydrates and potentially higher intake than healthy peers, consistent with findings from other studies [13, 14]. Additionally, in patients ≥ 15 years, carbohydrate intake tends to decline as fat intake rises, especially in those with higher phenylalanine levels, who also show greater fat consumption and about 30% present body mass index (BMI) above normal or the 75th percentile [15]. High refined carbohydrate and sugar intake has been associated with elevated glucose and triglycerides in healthy individuals [16], and probably with increased homeostasis model assessment for insulin resistance (HOMA‐IR) in PKU, particularly in overweight or obese patients [17].

Lipotoxicity contributes to insulin resistance in various metabolic disorders but its role in PKU remains poorly understood [18]. Persistently elevated phenylalanine concentrations resulting from poor adherence to a phenylalanine‐restricted diet may impair insulin regulation, reduce glucose utilization, and promote the development of insulin resistance [19]. Anthropometric measures such as waist circumference, waist‐to‐hip ratio, or waist‐to‐height ratio, in addition to BMI, are advisable to detect abnormal fat mass deposition [20].

Elevated total cholesterol and low‐density lipoprotein cholesterol (LDL‐C) levels during childhood increase cardiovascular risk in adulthood, alongside preclinical markers of atherosclerosis [19]. However, some studies have reported that patients with PKU following a phenylalanine‐restricted diet exhibit lower total cholesterol than healthy controls, possibly due to impaired cholesterol synthesis from down regulation of 3‐hydroxy‐3‐methylglutaryl coenzyme‐A reductase expression [21].

Patients with PKU should be encouraged to exercise [22]. Several studies suggest that when combined with dietary management, physical activity provides comparable health benefits as in the general population [23].

This study aimed to evaluate and compare dietary patterns and physical activity levels between children with different PKU phenotypes and matched healthy controls, and to assess the implications of these factors for their nutritional status.

## 2 Materials and Methods

### 2.1 Subjects and Study Design

This cross‐sectional observational study was conducted from August 2022 to December 2023. The study population comprised children aged 3 to 17 years, diagnosed with either mild hyperphenylalaninemia (HPA) or PKU through newborn screening and genetic confirmation. Participants were required to have no language barriers or comprehension difficulties. All participants were managed at the Congenital Metabolic Disease Unit of Sant Joan de Déu Hospital and attended regular clinical appointments.

A total of 82 children (46 girls and 36 boys) were recruited for the study. Among these, 22 had classic PKU (cPKU), all following a low natural protein (LP) diet supplemented with PS. Another 21 participants were PKU tetrahydrobiopterin responders (PKU‐BH_4_), with 10 on an LP diet receiving PS supplementation and 11 on a normal protein (NP) diet due to their phenylalanine tolerance. Additionally, 19 participants were diagnosed with HPA and followed a free dietary pattern. To ensure comparability, 20 healthy control participants from the same geographic region were included. These individuals were matched to the PKU patients by age (±1 year), sex, socioeconomic status, and educational level, differing only in the absence of the PKU condition.

The study received approval from the Research Ethics Committee of the Fundació de Recerca Sant Joan de Déu (codeCEImPIC‐163‐21). The parents or legal caregiver for all children, including controls, provided their written informed consent before their inclusion in the study.

### 2.2 Dietary Assessment

Parents completed a 4‐day food record for their children, which included two non‐working days (i.e., Saturday and Sunday). A trained metabolic dietitian used the nutrient analysis software ODIMET [10, 24] to calculate the nutritional intake based on the 4‐day food record.

Additionally, the KIDMED Mediterranean Diet Adherence Questionnaire with 16 questions was administered [25]. Children with HPA and PKU‐BH_4_ on an NP diet, and healthy controls answered all the questions on the questionnaire. In contrast, participants with cPKU and PKU‐BH_4_ who followed an LP diet responded to only eight tailored questions to assess adherence to a healthy eating pattern and were not required to respond to those questions related to high‐protein foods (related to fish, fast food, pulses, whole‐grain pasta or rice or cereals, nuts and dairies).

### 2.3 Anthropometric Parameters

Weight and length were measured using a combined stadiometer and digital weight measuring station (Seca 284, Seca, Hamburg, Germany). Height was recorded to the nearest 0.1 cm and weight to the nearest 0.1 kg. These anthropometric measurements were then converted to age‐ and sex‐specific *z*‐scores using the SEGHNP Pediatric Nutrition Application (available at https://www.seghnp.org/nutricional), which is based on the Barcelona Longitudinal Growth Study 1995–2017 [26].

### 2.4 Physical Activity Assessment

Physical activity was assessed using two age‐specific instruments: the validated Assessment of Physical Activity Levels Questionnaire (APALQ) [27] for children aged 9–18 years, and a tailored questionnaire for those aged 3–8 years (Table S1). While the APALQ evaluates weekly activity through categorical responses, the custom tool records specific weekly hours dedicated to sports and structured play. Both questionnaires utilize a cumulative scoring system (maximum 22 points) where higher values reflect greater activity levels, based on metabolic equivalent tables for pediatric populations [28]. For the custom questionnaire, frequency and duration were converted into a standardized score: “Once a week” corresponded to 1–1.5 h; “2–3 times a week” to 2 or 3 h; and “≥ 4 times a week” to at least 4 h (Table S1). School‐based activity and family involvement were also scored similarly. Participants scoring 2–9 points were classified as “lightly active,” those with 10–15 points as “moderately active,” and those with > 15 points as “very active.”

### 2.5 Biochemical Analysis

Blood samples were collected by venipuncture after a fasting period of at least 12 h. The following plasma parameters were determined: total cholesterol; high‐density lipoprotein cholesterol (HDL‐C); low‐density lipoprotein cholesterol; triglycerides; and fasting glucose. Amino acids, including phenylalanine, tyrosine, L‐Alanine, L‐Carnitine, and Tryptophan, were also measured. The iron profile consisted of iron, transferrin, and ferritin levels. Additionally, levels of vitamins and minerals, such as vitamin A, B_12_, D, E, folic acid, zinc, and selenium were determined. The composition of polyunsaturated fatty acids (PUFAs), in erythrocytes, including linoleic acid, α‐linolenic acid, arachidonic acid, eicosapentaenoic acid, and docosahexaenoic acid (DHA) was also analyzed.

Insulin levels were measured using solid‐phase enzyme‐labeled chemiluminescent immunometric assay. The HOMA‐IR, the triglyceride‐glucose index, and the triglyceride‐to‐HDL cholesterol ratio were calculated using the standard formulas [29, 30, 31].

### 2.6 Statistical Analyses

Statistical analyses were performed using SPSS version 27.0 software (SPSS Inc., Chicago, IL, USA). Normality was assessed using the Kolmogorov–Smirnov test, and homogeneity of variance was assessed using Levene's test. The Kruskal–Wallis and Mann–Whitney non‐parametric tests were employed to compare the dietary intake of energy and nutrients (including proteins, total fat, SFAs, monounsaturated fatty acids (MUFAs), PUFAs, carbohydrate, sugars, fiber, starch, and cholesterol) among participant phenotype groups. Results are presented as medians with interquartile ranges.

Anthropometric parameters and plasma biochemical data were transformed using natural logarithms due to their skewed distributions. Data were described using means and standard deviations (SD) and analyzed using ANOVA with Bonferroni correction. Pearson's correlation was used to examine the relationship between variables. Multivariate linear regression models were used to associate dietary intake and clinical variables. Participants with PKU were stratified for their dietary treatment: LP (*n* = 30) or NP (*n* = 32) (Supporting Information). Multivariate linear regression models were performed to examine the associations between plasma biochemical parameters and dietary treatment among PKU participants, stratified by LP or NP, and compared with the control group. In addition to the dichotomous classification, natural protein intake (%) was also modeled as a continuous variable to explore potential dose–response relationships. All models were adjusted for relevant covariates, including age, sex, weight, height, physical activity and macronutrients intake (%). Participants were further categorized in tertiles of HOMA‐IR. Differences across tertiles and dietary treatments were analyzed using Kruskal–Wallis and Mann–Whitney post hoc test (dietary data), or ANOVA with Bonferroni correction (biochemical and anthropometric data), as appropriate. A *p*‐value of < 0.05 was considered statistically significant.

## 3 Results

A total of 82 children, with a median age of 10.0 ± 4.1 years (56% female), were recruited for this study. Tables 1 and 2, and Tables S2 and S3, present findings stratified by PKU phenotype and control group.

**TABLE 1:** The table containing data on anthropometric, biochemical, and physical activity measurements of 62 participants with three phenotypes of PKU and control subjects.

Evaluated parameters^1^	cPKU (*n* = 22)	PKU—BH_4_ (*n* = 21)	HPA (*n* = 19)	Controls (*n* = 20)	*p*
Age, years	11.0 ± 4.1	9.8 ± 4.4	8.7 ± 3.8	10.3 ± 3.8	0.32
Females, *n* (%)	12 (55)	13 (62)	12 (63)	9 (50)	0.64
Anthropometric parameters					
Weight *Z*‐score	0.9 ± 1.1	0.2 ± 1.4	0.1 ± 1.3	0.4 ± 1.2	0.81
Height *Z*‐score	−0.5 ± 1.1	−0.1 ± 1.0	0.1 ± 1.4	0.0 ± 0.9	0.39
BMI *Z*‐score	0.6 ± 1.2	0.3 ± 1.3	0.6 ± 1.2	0.6 ± 1.5	0.54
Plasma lipid profile, glycemic markers, and insulin resistance parameters					
Total cholesterol, mg/dL	143 ± 29	149 ± 22	156 ± 27	156 ± 20	0.27
LDL‐C, mg/dL	75 ± 22^a^	82 ± 18^ab^	86 ± 20^ab^	91 ± 16^b^	0.042
HDL‐C, mg/dL	54 ± 11	55 ± 11	61 ± 14	54 ± 10	0.18
Non‐HDL‐C, mg/dL	90 ± 24	94 ± 20	96 ± 20	102 ± 17	0.26
Triglycerides, mg/dL	65 ± 23	55 ± 26	55 ± 20	55 ± 17	0.23
Glucose, mg/dL	88 ± 11	83 ± 7	85 ± 7	87 ± 7	0.27
Insulin, mU/L	5.1 ± 3.6	5.3 ± 4.3	3.4 ± 3.3	5.2 ± 5.0	0.51
HOMA‐IR	1.1 ± 0.8	1.1 ± 0.9	0.8 ± 0.6	1.2 ± 1.3	0.46
Triglyceride/HDL‐C ratio	1.3 ± 0.5	1.1 ± 0.7	1.0 ± 0.4	1.1 ± 0.5	0.38
Triglyceride‐glucose index	4.3 ± 0.2	4.2 ± 0.2	4.2 ± 0.2	4.2 ± 0.1	0.13
Plasma amino acid profile					
Phenylalanine, μmol/L	376 ± 176^a^	360 ± 244^a^	213 ± 79^b^	59 ± 12^c^	< 0.001
Tyrosine, μmol/L	46 ± 15^b^	49 ± 14^b^	60 ± 10^a^	67 ± 13^a^	< 0.001
Phenylalanine/tyrosine ratio	8.8 ± 4.7^a^	8.4 ± 7.0^a^	3.7 ± 1.6^b^	0.8 ± 0.1^c^	< 0.001
Adequate metabolic control^2^	19 (86)	18 (86)	19 (100)	—	0.23
Physical activity evaluation					
Physical activity score	14 (10–16)^b^	13 (11–15)^b^	15 (11–16)^b^	16 (12–19)^a^	0.015

Abbreviations: BMI, body mass index; cPKU, classic phenylketonuria; HDL‐C, high‐density lipoprotein cholesterol; HOMA‐IR, homeostatic model assessment insulin resistance; HPA, mild hyperphenylalaninemia; LDL‐C, low‐density lipoprotein cholesterol; non‐HDL‐C, non‐high‐density lipoprotein cholesterol; PKU‐BH_4_, phenylketonuria with tetrahydrobiopterin treatment.

^1^ All values are mean (SD) or *n* (%) or median (IQR), as appropriate. Means in a row with superscripts without a common letter differ, *p* < 0.05. Data analyzed by one‐factor ANOVA for continuous variables, chi‐square test, or Kruskal–Wallis and Mann–Whitney post hoc test, as appropriate.

^2^ Adequate metabolic control: < 12 years 120–360 μmol/L; > 12 years 360–600 μmol/L.

**TABLE 2:** Energy and macronutrient intake from the total diet, energy and macronutrient intake from special low‐protein foods and energy and macronutrient intake from supplements in subjects with three PKU phenotypes and the control group.^1^

	Total dietary intake (including SLPFs and PS)				*p*	SLPFs Intake^2^		*p*	PS^2^		*p*
	cPKU (*n* = 22)	PKU—BH_4_ (*n* = 21)	HPA (*n* = 19)	CONTROL (*n* = 20)		PKU (*n* = 22)	PKU—BH_4_ (*n* = 21)		PKU (*n* = 22)	PKU—BH_4_ (*n* = 21)	
Energy, kcal/day	1896 (1632–2220)	1731 (1463–2029)	1596 (1453–1852)	1755 (1491–1842)	0.17	495 (348–768)	35 (0.0–119)	< 0.001	595 (456–761)	32 (0.0–409)	< 0.001
Energy, %	100	100	100	100	—	29 (18–37)	1 (0–6)	< 0.001	32 (26–36)	5 (0–26)	< 0.001
Total protein, %	15.1 (12.3–18.5)	13.9 (11.5–17.0)	14.7 (13.3–15.8)	15.0 (13.4–16.9)	0.75	0.4 (0.3–0.5)	0.01 (0.0–0.1)	< 0.001	12.0 (9.8–16.6)	1.9 (0.0–6.8)	< 0.001
Natural protein, %	2.6 (2.0–3.0)^c^	10.8 (5.3–13.5)^b^	14.7 (13.3–15.8)^a^	15.0 (13.4–16.9)^a^	< 0.001	—	—	—	—	—	—
Ratio total protein/100 kcal	3.8 (3.1–4.6)	3.5 (2.9–4.3)	3.7 (3.3–4.0)	3.8 (3.3–4.2)	0.76	—	—	—	—	—	—
Phenylalanine, mg/day	449 (360–526)^c^	1281 (760–2257)^b^	2052 (1364–2633)^a^	1615 (1369–1991)^ab^	< 0.001	92.6 (30.7–112.4)	1.0 (0.0–28.5)	< 0.001	0.0 (0.0–28.0)	0.0 (0.0–0.0)	0.001
Tyrosine, mg/day	6248 (4027–8612)^a^	1031 (774–4110)^b^	791 (656–1334)^c^	712 (605–841)^c^	< 0.001	5.3 (0.0–9.3)	0.0 (0.0)	< 0.001	5993 (3915–8379)	0.0 (0.0–3017)	< 0.001
Carbohydrates, %	55.2 (51.5–58.7)^a^	48.2 (44.5–53.1)^b^	47.5 (45.0–49.6)^b^	47.5 (44.2–50.7)^b^	< 0.001	23.0 (13.9–37.7)	1.0 (0.0–5.3)	< 0.001	11.2 (7.3–15.3)	2.2 (0.0–8.7)	0.001
Sugars, %	18.4 (14.8–22.5)^a^	11.7 (8.7–17.5)^bc^	9.7 (8.7–13.0)^b^	13.7 (11.7–18.4)^c^	< 0.001	3.1 (1.4–6.3)	0.0 (0.0–1.2)	< 0.001	5.3 (01.9–6.6)	1.1 (0.0–3.3)	< 0.001
Fiber, g/day	23.7 (20.5–30.2)^a^	15.8 (11.6–24.6)^b^	11.9 (10.0–16.7)^c^	14.3 (10.4–16.9)^bc^	< 0.001	9.2 (3.3–12.7)	0.0 (0.0–0.8)	< 0.001	0.9 (0.0–9.0)	0.0 (0.0–0.0)	0.052
Starch, g/day^3^	20.0 (12.9–32.5)	23.1 (15.0–31.0)	24.2 (18.2–41.7)	15.6 (8.7–25.3)	0.27	—	—	—	2.1 (0.0–6.9)	0.0 (0.0–3.8)	0.19
Total fats, %	31.0 (27.0–36.6)^b^	38.6 (32.7–43.5)^a^	38.7 (32.9–40.2)^a^	37.5 (33.0–40.2)^a^	0.014	5.5 (3.4–9.4)	0.1 (0.0–1.9)	< 0.001	6.1 (3.2–9.8)	0.1 (0.0–5.3)	0.04
SFAs, %	7.7 (7.0–11.0)	10.6 (7.4–11.5)	10.6 (9.6–12.4)	11.3 (9.0–12.6)	0.75	5.5 (2.5–10.4)	0.1 (0.0–1.5)	< 0.001	1.1 (0.6–1.8)	0.0 (0.0–0.8)	0.002
MUFAs, %	12.8 (9.6–16.4)^bc^	15.4 (12.6–19.5)^a^	13.6 (12.3–14.6)^ab^	11.6 (10.4–12.8)^c^	0.022	0.1 (0.0–0.7)	0.0 (0.0–0.0)	0.001	1.6 (0.2–4.9)	0.0 (0.0–2.3)	0.07
PUFAs, %	3.4 (2.3–4.3)^b^	4.5 (3.9–5.6) ^a^	3.5 (3.0–4.2) ^b^	3.9 (3.0–4.4) ^ab^	0.019	0.0 (0–0.3)	0.0 (0.0)	0.001	1.4 (0.4–2.2)	0.0 (0.0–1.3)	0.03
Cholesterol, mg/day^4^	7.3 (3.7–16.1)^d^	102 (31.9–225.3)^bc^	204 (131.2–296.2)^a^	165 (127.7–207.8)^ab^	< 0.001						

Abbreviations: MUFAs, monounsaturated fatty acids; PS, protein substitutes; PUFAs, polyunsaturated fatty acids; SFAs, saturated fatty acids; SLPFs, special low‐protein foods.

^1^ All values are median (IQR). Medians in a row with superscripts without a common letter differ, *p* < 0.05. Data analyzed by Kruskal–Wallis and Mann–Whitney post hoc test.

^2^ Patients with HPA or controls have not been included since they do not consume SLPFs and PS.

^3^ Data on starch are not available in SLPFs.

^4^ Data on cholesterol is not available in SLPFs and PS.

### 3.1 Characteristics of Study Participants by PKU Phenotypes and Control Group

No significant differences were observed among groups in terms of age, sex, anthropometric measurements, or biochemical markers for total cholesterol, glucose, triglycerides, insulin, or HOMA‐IR (Table 1). However, the control group exhibited significantly higher LDL‐C levels compared to those with cPKU (*p* = 0.042). Adequate metabolic control was achieved in 56 of 62 subjects (90%), with notably higher phenylalanine levels and phenylalanine/tyrosine ratio and lower tyrosine levels in the cPKU and PKU‐BH_4_ groups compared to the HPA group and controls.

Participants in the cPKU group also showed elevated plasma vitamin B_12_ and folic acid levels compared to the other groups, and higher plasma vitamin D compared to subjects in the HPA group (Table S2). cPKU also showed significantly higher levels of arachidonic acid than the HPA group and higher levels of DHA compared to subjects in the PKU‐BH_4_ and controls.

With regard to physical activity, the control group showed the highest activity levels, with scores significantly surpassing those of the PKU phenotypes (Table 1). Interestingly, no significant differences were observed among the three PKU phenotype groups themselves in the physical activity score (*p* = 0.47). In addition, when the three categories (lightly active, moderately active, or very active) were compared, no significant differences were observed among the three PKU phenotypes (Fisher's exact test, *p* = 0.13). However, significant differences emerged when comparing PKU participants and controls (Fisher's exact test, *p* = 0.018). Pairwise comparisons provided further insight into these differences: the cPKU group was significantly more “lightly active” than the control group (8/22, 36% vs. 1/20, 5%, respectively; *p* = 0.04), while the PKU‐BH_4_ group showed a significantly lower proportion of “very active” individuals compared to controls (1/21, 5% vs. 9/20, 45%, respectively; *p* = 0.004). In contrast, the HPA group did not differ significantly from the control group in any category (*p* = 0.25).

### 3.2 Assessment of Energy and Macronutrient Intake and Dietary Patterns Among PKU Phenotypes and Control Group Participants

There were no significant differences in total energy, total protein, SFAs, and starch intake between PKU participants and controls (Table 2). However, both cPKU and PKU‐BH_4_ subjects consumed a lower percentage of natural protein (PRO) and phenylalanine compared to the HPA and control groups, with tyrosine intake significantly higher (*p* < 0.001), attributable to PS intake. Carbohydrate and sugar intakes were also higher in cPKU subjects than in other groups. The 42% and 20% of carbohydrate and 17% and 29% of free sugars in the cPKU group derived from SLPFs and PS, respectively. Fiber intake was highest among the cPKU group, with a significant descending trend across the PKU‐BH_4_ and HPA groups (*p* < 0.001), reflecting the extent of the LP dietary treatment.

Participants with cPKU consumed the lowest amount of total fats (Table 2). No significant differences were found between the groups in the SFAs. It's noteworthy that a high proportion of SFAs in the cPKU group (71%) come from the SLPFs, while in the PKU‐BH_4_ group, they come from natural foods. In addition, 41% of PUFAs in the cPKU group come from PS.

The global KIDMED questionnaire answered only by PKU participants with non‐restricted diet (Table S3) indicated that 53% of the HPA group and 45% of the PKU‐BH_4_ group following an NP diet treatment demonstrated a need to improve in dietary patterns, compared to only 10% in the control group. When examining the eight specific items from the KIDMED questionnaire available for comparison between the PKU groups and the control group, no significant differences were observed, except in the item regarding consumption of sweets and candies several times per day. In this case, 36% of cPKU patients reported such frequent consumption, compared to 0% in the control group (*p* = 0.001). No significant differences were found in the use of olive oil or intake of fruits and vegetables. However, while not statistically significant, 27% of subjects with PKU reported skipping breakfast, compared to 5% in the control group.

### 3.3 Study of the Relationship Between Carbohydrate, Total Fat, and SFA Consumption and Implication for Nutritional Status and Sex Effects

Our results show that a large percentage of carbohydrates and fats in the cPKU group come from SLPFs and PS, so we investigated whether there is a correlation between the intake of these nutrients and some biochemical parameters (Table S4).

In the cPKU group, total fat intake was positively correlated with both LDL‐C and HDL‐C levels (Table S4). Given the potential role of phenylalanine in modulating hepatic 3‐hydroxy‐3‐methylglutaryl coenzyme‐A reductase activity, additional correlation analyses were performed adjusting for phenylalanine levels (μmol/L). After adjustment, the positive association between total fat intake and HDL‐C remained statistically significant (*β* = 0.98; 95% CI: 0.31–1.65; *p* = 0.007), while the association with LDL‐C showed a positive trend that did not reach statistical significance (*β* = 1.42; 95% CI: −0.12 to 2.97; *p* = 0.069). In the PKU‐BH_4_ group, total fat intake was negatively correlated with insulin and HOMA‐IR, while SFA intake was positively correlated with TC, LDL‐C, and non‐HDL‐C levels (*p* < 0.05). After adjusting for age, the inverse association between total fat intake and insulin remained statistically significant (*β* = −0.21; 95% CI: −0.41 to −0.02; *p* = 0.035) whereas the association with HOMA‐IR showed a similar trend but did not reach statistical significance (*β* = −0.04; 95% CI: −0.09 to 0.00; *p* = 0.052). No significant correlations were observed between carbohydrate intake and any of the analytical parameters (Table S4). When the three different PKU phenotypes were analyzed together (*n* = 62), carbohydrate intake was positively correlated with HOMA‐IR (*r* = 0.29, *p* < 0.05), insulin (*r* = 0.30, *p* < 0.05), triglycerides (*r* = 0.26, *p* < 0.05). However, after adjusting for age, these associations were no longer significant, with only a trend remaining for insulin and HOMA‐IR (*p* = 0.06 and 0.08, respectively). In addition, these correlations persisted in females [HOMA‐IR (*r* = 0.43, *p* < 0.001), insulin (*r* = 0.46, *p* < 0.001), triglycerides (*r* = 0.40, *p* < 0.05)] but were not observed in males or in the male and female control groups (*p* > 0.05). After adjusting for age, the association remained only as a trend for insulin (*β* = 0.28; *p* = 0.087) and was no longer significant for HOMA‐IR (*β* = 0.20; *p* = 0.18).

### 3.4 Characteristics of Study Participants, Dietary Intake, and Plasma Biochemical Parameters by Tertiles of HOMA‐IR

Based on the findings related to carbohydrate and sugar consumption in the PKU groups, we stratified participants with PKU into HOMA‐IR tertiles (Table 3) to explore potential relationships between dietary intake and insulin resistance and compared these results with those of the control group.

**TABLE 3:** Anthropometric parameters, energy and macronutrient intake stratified by HOMA‐IR tertiles in different PKU phenotypes and the control group.^1^

	Tertiles of HOMA‐IR^4^ in PKU participants				*p*
	1 < 0.43 (*n* = 20)	2 0.44–1.13 (*n* = 19)	3 > 1.17 (*n* = 20)	Control group 0.21–5.86 (*n* = 19)	
cPKU, *n* (%)	5 (25)	10 (53)	7 (35)	—	< 0.001
PKU‐BH_4_, *n* (%)	8 (40)	5 (26)	8 (40)	—	
HPA, *n* (%)	7 (35)	4 (21)	5 (25)	—	
Age, years	7.7 ± 4.3^b^	10.4 ± 3.2^ab^	11.4 ± 4.2^a^	10.5 ± 3.9^ab^	0.027
Females, *n* (%)	10 (50)	11 (58)	14 (70)	9 (47)	0.52
Anthropometric parameters					
Weight *Z*‐score	0.2 ± 1.0^ab^	−0.5 ± 0.9^b^	0.5 ± 1.3^a^	0.4 ± 1.2^ab^	0.017
Height *Z*‐score	0.1 ± 1.2	−0.4 ± 0.9	−0.3 ± 1.3	−0.2 ± 0.9	0.48
BMI *Z*‐score	0.2 ± 1^ab^	−0.2 ± 1.0^b^	0.9 ± 1.4^a^	0.7 ± 1.3^ab^	0.021
Energy and macronutrient intake					
Energy, kcal/day	1546 (1314–1924)	1832 (1641–2183)	1806 (1566–2143)	1772 (1573–1842)	0.30
Total protein, %	13.8 (11.9–16.1)^b^	14.2 (11.5–16.0)^b^	16.6 (14.2–18.9)^a^	14.9 (13.3–14.9)^ab^	0.040
Natural protein, %	11.1 (4.3–13.9)^b^	3.7 (2.3–13.0)^b^	8.2 (3.2–14.2)^b^	14.9 (13.3–16.7)^a^	< 0.001
Phenylalanine, mg/day	1211 (534–2338)^a^	596 (474–1411)^b^	990 (517–1946)^a^	1612 (1369–1901)^a^	0.029
Tyrosine, mg/day	815 (661–3221)^b^	3925 (1206–6019)^a^	3347 (65–6582)^a^	727 (625–841)^b^	< 0.001
Carbohydrates, %	47.5 (44.0–52.0)^ab^	51.5 (48.0–54.3)^a^	52.6 (47.5–55.6)^a^	47.2 (44.2–50.7)^b^	0.03
Sugars, %	12.8 (9.0–17.9)	17.4 (10.9–20.8)	13.4 (10.4–19.2)	13.8 (12.5–18.3)	0.65
Fiber, g/day	15.4 (10.7–19.6)	21.4 (13.5–28.8)	16.4 (12.0–23.8)	14.5 (11.8–16.9)	0.06
Starch, g/day^2^	24.1 (18.9–36.8)	22.8 (10.2–36.9)	19.0 (12.9–29.8)	11.0 (8.7–25.3)	0.33
Total fat, %	40.2 (29.0–42.6)	36.0 (31.6–38.3)	34.8 (26.9–38.6)	38.1 (33.5–40.2)	0.27
SFAs, %	10.2 (8.9–12.1)	10.3 (7.8–13.6)	11.2 (7.4–13.2)	11.4 (9.0–12.6)	0.99
MUFAs, %	14.3 (12.3–17.7)	15.4 (10.3–18.1)	13.3 (11.0–15.1)	11.7 (10.5–12.8)	0.10
PUFAs, %	3.6 (3.0–4.7)	4.3 (3.5–5.0)	3.5 (2.7–4.2)	3.9 (3.0–4.4)	0.34
Cholesterol, mg/day^3^	94.0 (29.2–199.5)^b^	26.5 (5.9–229.5)^b^	86.3 (16.1–197.1)^b^	163 (127.7–207.8)^a^	0.05
KIDMED					
Adapted KIDMED score	4 ± 1	4 ± 1	3 ± 1	4 ± 1	0.50
Physical activity evaluation					
Physical activity score	12 (10–15)^b^	15 (12–17)^ab^	14 (10–15)^b^	16.0 (13.0–20.0)^a^	0.001

Abbreviations: MUFAs, monounsaturated fatty acids; PFAAs, precursor‐free L‐amino acid supplement; PUFAs, polyunsaturated fatty acids; SFAs, saturated fatty acids; SLPFs, special low‐protein foods.

^1^ All values are median (IQR) or *n* (%), as appropriate. Medians in a row with superscripts without a common letter differ, *p* < 0.05. Data analyzed by Kruskal–Wallis and Mann–Whitney post hoc test.

^2^ Data on starch is not available in SLPFs.

^3^ Data on cholesterol is not available in SLPFs and PFAAs.

^4^ HOMA‐IR tertiles have been calculated in the individuals with PKU.

Among individuals with PKU, participants in tertile 3 were significantly older than those in tertile 1 and participants in tertile 3 showed higher weight and BMI Z‐scores compared to those in tertile 2 of HOMA‐IR. Among individuals with PKU, those in the highest tertile of HOMA‐IR exhibited a higher percentage of total protein intake compared to other PKU groups but not compared to the control group. No significant differences in natural protein and carbohydrate intakes were observed across HOMA‐IR tertiles of PKU. No significant differences in KIDMED score and its specific questions were observed between the tertiles. Homogeneous physical activity levels were observed across the HOMA‐IR tertiles within the PKU groups. Statistically significant differences in physical activity were found between the first and third HOMA‐IR tertiles of PKU with the control group and a trend was observed between the second HOMA‐IR tertile and the control group (*p* = 0.074).

From the lowest to the highest HOMA‐IR tertile, participants exhibited progressively higher levels of insulin (T1: 1.6 ± 0.6 mU/L; T2: 4.0 ± 0.9 mU/L; T3: 9.1 ± 3.2 mU/L) and HOMA‐IR (T1: 0.3 ± 1.2; T2: 0.9 ± 0.2; T3: 1.9 ± 0.7).

### 3.5 Evaluation of Dietary Intake, Anthropometric and Biochemical Parameters According to Natural Protein Tolerance

Given the heterogeneity in PKU phenotypes and dietary treatments in our sample, we classified participants based on dietary protein restriction (LP and NP) (Table 4, Tables S5 and S6). No significant differences in anthropometric data, sex, or age were observed among the groups. Participants in the LP diet had significantly higher percentages of carbohydrate, sugars, tyrosine, and fiber intake and lower intake of natural protein, phenylalanine, and total fat intakes than those in NP. Regarding biochemical parameters, adherence to a LP was independently associated with lower plasma levels of TC, HDL‐C, and tyrosine compared to participants on NP. Conversely, participants on a LP were positively associated with higher levels of vitamin B_12_, folates, α‐linolenic acid, DHA, and eicosapentaenoic acid. Sensitivity analyses were done using associations with % of natural protein (Table S7). Higher natural protein intake was associated with lower plasma concentrations of several micronutrients—including DHA, folic acid, vitamins D and B_12_, and zinc—along with reduced phenylalanine levels and a lower phenylalanine/tyrosine ratio, but higher total cholesterol, LDL‐C, and tyrosine.

**TABLE 4:** Multivariate linear regression models for significant association between plasma biochemical parameters and dietary treatment in PKU participants with and without low‐protein diet treatment and the control group.

	PKU normal diet—NP [ref: Control] *β* (95% CI)	PKU Low natural protein diet—LP [ref: Control] *β* (95% CI)	PKU Low natural protein diet – LP [ref = PKU normal diet] *β* (95% CI)
Plasma lipid profile, glycemic markers, and insulin resistance parameters			
Total Cholesterol, mg/dL	−0.34 (−13.95–13.28)	−18.00 (−33.1–−2.90)*	−17.66 (−31.74–−3.59)*
LDL‐C, mg/dL	−6.04 (−16.46–4.38)	−14.81 (−26.37–−3.26)*	−8.78 (−19.55–2.00)
HDL‐C, mg/dL	6.47 (0.05–12.89)*	−2.95 (−10.07–4.17)	−9.43 (−16.07–−2.79)**
Non‐HDL‐C, mg/dL	−6.81 (−18.04–4.42)	−15.05 (−27.51–−2.59)*	−8.24 (−19.85–3.37)
Triglycerides, mg/dL	−0.39 (−12.91–12.13)	−2.40 (−16.29–11.49)	−2.01 (−14.95–10.94)
Triglyceride/HDL‐C ratio	−0.12 (−0.42–0.19)	0.01 (−0.33–0.35)	0.13 (−0.19–0.45)
Triglyceride‐glucose index	−0.03 (−0.13–0.08)	−0.03 (−0.14–0.08)	0.00 (−0.11–0.10)
Glucose, mg/dL	−1.47 (−6.51–3.57)	−0.31 (−5.9–5.28)	1.16 (−4.05–6.37)
Insulin, mU/L	−0.14 (−2.45–2.16)	−0.34 (−2.9–2.22)	−0.19 (−2.58–2.19)
HOMA‐IR	0.08 (−0.48–0.64)	−0.14 (−0.75–0.47)	−0.21 (−0.78–0.35)
Plasma vitamins and minerals			
Vitamin A, μmol/L	−0.10 (−0.37–0.16)	0.06 (−0.23–0.35)	0.16 (−0.11–0.44)
Vitamin B12, pmol/L	−43.06 (−161.23–75.11)	365.28 (234.17–496.39)***	408.33 (286.13–530.53)***
Vitamin D, ng/mL	26.54 (19.96–33.12)***	35.34 (28.04–42.64)***	8.80 (1.99–15.60)*
Vitamin E, μmol/L	−1.90 (−7.53–3.73)	−0.27 (−6.52–5.98)	1.63 (−4.20–7.45)
Folic acid, nmol/L	1.36 (−2.89–5.61)	17.58 (12.86–22.29)***	16.22 (11.82–20.61)***
Zinc, μg/L	132.89 (−61.65–327.43)	195.23 (−20.61–411.08)	62.34 (−138.83–263.52)
Selenium, μg/L	−0.16 (−9.74–9.41)	−3.63 (−14.26–6.99)	−3.47 (−13.37–6.44)
Polyunsaturated fatty acids			
Linoleic acid, nmol/g Hb	50.61 (−422.72–523.93)	764.78 (239.63–1289.93)**	714.17 (224.71–1203.63)**
α‐Linolenic acid, nmol/g Hb	−2.29 (−7.14–2.57)	8.55 (3.16–13.94)**	10.84 (5.82–15.86)***
Arachidonic acid, nmol/g Hb	−76.31 (−787.97–635.36)	578.36 (−211.23–1367.95)	654.66 (−81.27–1390.59)
Eicosapentaenoic acid, nmol/g Hb	2.54 (−15.44–20.52)	31.7 (11.76–51.65)**	29.17 (10.58–47.76)**
Docosahexaenoic acid, nmol/g Hb	8.40 (−192.68–209.48)	466.11 (243.01–689.21)***	457.71 (249.78–665.65)***
Iron profile			
Iron, μmol/L	5.13 (0.87–9.39)*	1.51 (−3.22–6.23)	−3.63 (−8.03–0.78)
Transferrin, mg/L	1140.66 (625.2–1656.12)***	718.84 (146.94–1290.74)*	−421.83 (−954.86–111.21)
Ferritin, μg/L	15.86 (1.75–29.98)*	19.64 (3.97–35.3)*	3.77 (−10.83–18.37)
Plasma amino acid profile			
Phenylalanine, μmol/L	188.89 (97.97–279.82)***	345.51 (244.63–446.38)***	156.61 (62.59–250.64)**
Tyrosine, μmol/L	−9.27 (−16.87–−1.67)*	−29.24 (−37.67–−20.81)***	−19.97 (−27.83–−12.11)***
Alanine, μmol/L	−46.01 (−83.74–−8.29)*	−64.97 (−106.83–−23.11)**	−18.95 (−57.97–20.06)
L‐carnitine, μmol/L	−5.64 (−11.34–0.06)	1.62 (−4.71–7.94)	7.26 (1.37–13.16)*
Tryptophan, μmol/L	−6.98 (−13.72–−0.24)*	−6.71 (−14.19–0.76)	0.27 (−6.70–7.24)
Phenylalanine/tyrosine ratio	3.52 (0.96–6.08)**	9.84 (7.00–12.68)***	6.32 (3.67–8.97)***

*Note:* Models are adjusted for sex, age (years), weight (kg), height (cm), physical activity, and macronutrient intake (%).

Abbreviations: HDL‐C, high‐density lipoprotein cholesterol; LDL‐C, low‐density lipoprotein cholesterol; non‐HDL‐C, non‐high‐density lipoprotein cholesterol.

* *p* < 0.05.

** *p* < 0.01.

*** *p* < 0.001.

## 4 Discussion

This study investigates the quality of diet in addition to physical activity in children with different PKU phenotypes to understand the impact on their nutritional status, comparing them with healthy controls.

Our cross‐sectional data show no significant differences in age, sex, and anthropometric measures (weight, height, or BMI) between groups and are consistent with other recent studies [13, 32]. Importantly, body composition was not assessed in our study. Ilgaz et al. [33] concluded in their systematic review, which involved children with PKU born before the 1990s, that there is an impairment in linear growth, particularly during early childhood and adolescence. Our findings do not allow conclusions on growth trends over time. Therefore, longitudinal research is needed to better understand how phenotype, dietary practices, and metabolic control influence growth and development in this population.

The diet of patients with PKU is guided by their individual phenylalanine/protein tolerance, which refers to the amount of phenylalanine and natural protein they can safely consume while maintaining metabolic control. Pinto et al. [34], concluded that HPA and tetrahydrobiopterin responders can tolerate more natural protein than those with cPKU, a finding in line with our results (2.6% in PKU vs. 10.8% in PKU‐BH_4_ vs. 14.7% in HPA). Other studies [35] show that their diet, which includes SLPFs and varying PS intake, is higher in carbohydrate and sugars and lower in fat compared to other PKU phenotypes with more natural protein intake. This matches our results that present a higher carbohydrate and sugar intake. The frequent consumption of sweets and candies, as indicated by the KIDMED results, along with the percentage contributed by SLPFs and PS, could likely explain the dietary patterns observed. The KIDMED score indicates that 53% of the HPA and 45% of the PKU‐BH_4_ group on an NP diet need to improve the quality of their diet, compared to 10% in the control group.

We observed a significantly higher tyrosine intake in the cPKU group, explained by strict dietary management that limits natural protein and relies on tyrosine‐enriched protein substitutes to compensate for impaired phenylalanine to tyrosine conversion. In contrast, BH_4_‐responsive PKU and HPA patients follow less restrictive diets and retain partial enzyme activity, enabling more efficient endogenous tyrosine synthesis and reducing the need for supplementation [7, 22].

In our study, carbohydrate and sugar intake was higher in the cPKU group compared to the PKU‐BH_4_ and HPA groups. High sugar consumption has been associated to alterations in the biochemical profile, including glucose and triglyceride levels [14]. HOMA‐IR and the triglyceride‐glucose index are the most commonly used indices to assess insulin resistance [36]. In several studies, the HOMA‐IR positively correlated with age, BMI, and pubertal stage, although not with sex [37, 38, 39]. Couce et al. [17] demonstrated that fasting insulin levels and HOMA‐IR were significantly higher in children with PKU compared to healthy controls, with significant differences observed based on diagnosis and phenylalanine tolerance, probably related to their higher caloric intake in the form of carbohydrate content. We found significant differences in the age, BMI and weight of participants between the first and third tertiles of HOMA‐IR, which could be related to pubertal status [37], results coinciding with other studies [38, 39]. Our study shows that SFA intake was positively correlated with HOMA‐IR and insulin in the cPKU group. In these individuals, 74% of total SFA intake originated from SLPFs. Our previous work has already demonstrated that SLPF products such as cheese, bread, pasta, and rice contain a higher proportion of SFA compared to their regular counterparts [10]. In addition, we found a positive correlation between carbohydrate intake and triglyceride levels, insulin concentrations, and HOMA‐IR in individuals with PKU, however after adjusting by age these associations disappeared, with only a trend remaining for insulin. Although the correlation between carbohydrate intake and insulin resistance weakens after adjusting for age, the trend, especially in girls, suggests the need for monitoring as they grow older. Adult women with PKU and poor dietary adherence show elevated insulin and HOMA‐IR levels [17]. Additionally, HOMA‐IR tends to increase with age in the general female population, reinforcing the need for long‐term follow‐up [40]. However, several studies have reported a higher prevalence of overweight and obesity among girls and women with PKU compared to boys [41, 42, 43], therefore, it is needed to highlight the importance of systematically evaluating potential contributing factors during routine clinical follow‐up visits as quality of diet. Body composition measures such as waist circumference could have helped detect to identify abnormal fat mass deposition in these individuals [20]. Currently, 86% of participants with PKU have good metabolic control, periodic monitoring phenylalanine levels within range is important, as persistently elevated phenylalanine concentrations, can impair insulin regulation, thereby reducing glucose utilization and promoting the development of insulin resistance [19]. In addition to monitoring phenylalanine levels, the distribution of body fat plays a critical role in assessing metabolic risk in individuals with PKU [44]. Visceral adiposity, particularly abdominal fat, is more strongly associated with insulin resistance [45], underscoring the importance of incorporating anthropometric measures such as waist circumference into routine clinical evaluations [20, 46]. Furthermore, it is essential to prevent the development of lipotoxicity, a condition characterized by the accumulation of lipids in non‐adipose tissues such as liver and skeletal muscle that can activate inflammatory pathways and oxidative stress, ultimately impairing insulin signaling and contributing to the onset of insulin resistance [47].

Although there are no differences in anthropometric variables or the lipid and glucose profiles, these results, in addition to the daily consumption of sweets and candies in the cPKU group, combined with lower activity levels could lead to an increased risk of overweight and obesity in children with PKU; values align with the general population [48, 49]. Although current scientific evidence does not conclusively support a direct association between a phenylalanine‐restricted diet and the development of overweight in individuals with PKU [50], good metabolic control, monitoring diet quality, and physical activity practice are essential to preventing the development of comorbidities associated with excess weight in PKU [20].

Dyslipidemia is a major contributor to atherosclerosis, prompting extensive investigation into lipoprotein profile alterations in PKU. Our findings revealed lower LDL‐C levels in cPKU compared to the control group, similar to other studies [51]. Furthermore, when stratifying the PKU population based on natural protein tolerance, individuals following a LP showed significantly lower levels of total cholesterol and HDL‐C compared to those with a NP. These results are consistent with previous studies reporting comparable lipid profile differences based on dietary treatment [52, 53]. These findings align with other authors' observations that these low cholesterol levels may result from a vegetable diet with a large consumption of fruits and vegetables [54, 55], an olive oil‐based diet and reduced cholesterol synthesis. A potential mechanism for this reduced synthesis is the decreased expression of 3‐hydroxy‐3‐methylglutaryl coenzyme A reductase, which might be influenced by elevated levels of phenylalanine and its metabolites [52, 53, 56]. PS provides not only amino acids but also essential vitamins, minerals, and PUFAs. Some studies found elevated vitamin levels, while others reported lower levels or no significant differences between PKU patients and healthy controls [57, 58, 59]. Our research indicates that vitamin B_12_ and folic acid levels were elevated in the cPKU compared to the PKU‐BH_4_, HPA, and control groups, which matches the type of diet, as participants with PKU and LP diet had high levels of these vitamins compared to those with NP diets. It is important to note that when adjusted for covariates, participants in the PKU LP diet group had higher levels of α‐linolenic acid, linoleic acid, eicosapentaenoic acid, and DHA than those participants in the PKU NP diet group. These differences underscore the importance of individualizing nutritional recommendations.

In children, physical activity is critical for motor development, cognitive improvement, psychosocial, and cardio‐metabolic health [60]. Children and young people who are physically active are more likely to continue the habit into adult life [61]. McDonald et al. [7] recommend that children with PKU engage in at least 30 to 45 min of daily physical activity to support overall health and optimize dietary management, taking into account the intensity of physical activity. Several studies suggest that when combined with dietary management, physical activity in PKU patients provides similar health benefits comparable to the general population [23] and the new European guidelines on the treatment of PKU recommend that participation in sports should be encouraged, always maintaining an adequate intake of nutrients and energy [20]. Our results indicate that cPKU and PKU‐BH_4_ participants are less active than the control group. Therefore, it is essential to continue encouraging children with PKU to engage in regular physical activity.

The concentration of phenylalanine in the blood is the primary determinant in treatment decisions [62]. Elevated phenylalanine levels, resulting from poor adherence to treatment, can lead to severe intellectual disability and nutritional deficiencies. Additionally, a phenylalanine‐restricted diet, high in carbohydrates and sugars, in patients with significant protein restrictions can result in weight gain and other associated comorbidities [15, 41, 42]. Eighty‐six per cent of cPKU and PKU‐BH_4_ participants and 100% of HPA subjects had good metabolic control.

Despite limitations such as a small sample size in global and for each phenotype, and the additional subdivision by sex, as well as the inherent challenges of working with a pediatric population, our homogeneous subject group enables meaningful comparisons, making our findings representative. Our study underscores the influence of diet quality in children with PKU and its health impacts, emphasizing the need for further research.

In conclusion, this study shows that carbohydrates and sugars intake was higher in the cPKU population compared with other PKU phenotypes and the control group, largely derived from special low‐protein foods and protein substitutes. Participants with PKU were less physically active than controls. Differences in lipid profiles between children with PKU following low‐ and normal‐protein diets suggest that dietary patterns alone may not fully explain these alterations, pointing to potential changes in cholesterol metabolism. Higher levels of vitamin B_12_, folic acid, and polyunsaturated fatty acids in children on low‐protein diets further highlight the need for individualized dietary and lifestyle management in PKU. Longitudinal studies are required to clarify the long‐term metabolic impact of phenotype, diet, and physical activity.

## Author Contributions

Study conception and design: Dolores Garcia‐Arenas, Aida Ormazabal, and Mireia Urpi‐Sarda. Data curation: Dolores Garcia‐Arenas and Paula Isern. Data collection: Dolores Garcia‐Arenas, Aida Ormazabal, and Paula Isern. Data analysis: Dolores Garcia‐Arenas, Aida Ormazabal, Paula Isern, Alba Tor‐Roca, and Mireia Urpi‐Sarda. Supervision: Aida Ormazabal, Paula Isern, Blanca Barrau‐Martinez, Arnau Gonzalez‐Rodriguez, Rafael Llorach, and Jaume Campistol‐Plana. Writing – original draft: Dolores Garcia‐Arenas and Mireia Urpi‐Sarda. Critically review and editing the manuscript: All authors.

## Funding

This work was supported by the project funded by the Fundació La Marató de TV3 (2020; Ref. 202014). It was also supported by project PID2024‐156020OB‐I00, funded by MICIU/AEI/10.13039/501100011033/FEDER, UE. B.B.‐M. was supported by a fellowship from the AGAUR‐Generalitat de Catalunya (2022 FI_B 01012). A.G.‐R. was supported by a contract from the project “Fundació La Marató de TV3, 2020”, by a fellowship from the AGAUR‐Generalitat de Catalunya (2024 FI‐1 00408) and by FPU 2024 contract from the Ministry of Science, Innovation and Universities (FPU23/03478).

## Ethics Statement

Clinical assessments and biomaterial reported in this paper were obtained in protocols approved by the Research Ethics Committee (CEIm) of the Fundació de Recerca Sant Joan de Déu (codeCEImPIC‐163‐21). All procedures followed were in accordance with the ethical standards of the committee responsible for human experimentation (institutional and national) and with the Helsinki Declaration of 1975, as revised in 2000.

## Consent

Parents provided written informed consent for participants.

## Conflicts of Interest

The authors declare no conflicts of interest.

## Supporting information


**Table S1:** Questionnaire on Guided Physical Activity Levels for children aged 3 to 8 years (adapted from Castro et al., 2019^2^)
**Table S2:** Biochemical parameters of subjects with the three phenotypes of PKU and control subjects^1^.
**Table S3:** Mediterranean Diet Quality Index according to PKU phenotype.
**Table S4:** Pearson's correlations of carbohydrate, total fat and SFA intake with biochemical parameters, stratified by PKU phenotype and control group.
**Table S5:** Anthropometric and plasma biochemical parameters stratified by dietary treatment in PKU participants with and without low‐protein diet treatment and the control group^1^.
**Table S6:** Energy and macronutrient intake stratified by dietary treatment in subjects with PKU and the control group^1^.
**Table S7:** Multivariate linear regression models for association between plasma biochemical parameters and natural protein intake (%).

## Data Availability

The data that support the findings of this study are available from the corresponding author upon reasonable request.
