# Supplementary material for: Comparative Analysis of Dietary Patterns in Children With Phenylketonuria Phenotypes and Controls: Implications for Nutritional Status
Source: J Inherit Metab Dis. 2026 May 3;49:e70196. doi: 10.1002/jimd.70196 (PMC13135907; doi:10.1002/jimd.70196)
Supplement: Supplementary file 1 — Table S1: Questionnaire on Guided Physical Activity Levels for children aged 3 to 8 years (adapted from Castro et al., 20192) Table S2: Biochemical parameters of subjects with the three phenotypes of PKU and control subjects1. Table S3: Mediterranean Diet Quality Index according to PKU phenotype. Table S4: Pearson's correlations of carbohydrate, total fat and SFA intake with biochemical parameters, stratified by PKU phenotype and control group. Table S5: Anthropometric and plasma biochemical parameters stratified by dietary treatment in PKU participants with and without low‐protein diet treatment and the control group1. Table S6: Energy and macronutrient intake stratified by dietary treatment in subjects with PKU and the control group1. Table S7: Multivariate linear regression models for association between plasma biochemical parameters and natural protein intake (%). [file JIMD-49-0-s001.docx]

**SUPPORTING INFORMATION**

**METHODOLOGY**

**Statistical Analysis**

In this work, we have used a dichotomous classification based on protein/phenylalanine tolerance to maintain plasma Phe levels within the therapeutic target range (restricted vs. unrestricted protein diet), a commonly applied approach in clinical research and practice ^1^ .

Participants were stratified into two groups according to their dietary treatment, which reflects phenotype severity and BH_4_ responsiveness. Individuals with classical PKU or BH_4_ non-responsiveness followed a strict low natural protein diet (LP) (n = 30), while those with milder phenotypes or BH_4_ responsiveness followed an unrestricted Phe diet or normal diet (NP) (n = 32).

**Supplementary Table S1.** Questionnaire on Guided Physical Activity Levels for children aged 3 to 8 years (adapted from Castro et al., 2019^2^ )

| No. | Question | Response Categories | Evaluation of the Answers* |
| --- | --- | --- | --- |
| 1 | In general terms, how many hours of physical activity does your child practice throughout the week? | 1: Never; 2: Less than 2 hours/week; 3: 2 to 4 hours/week; 4: 4 to 6 hours/week; 5: More than 6 hours/week | 1: +1; 2: +2; 3: +3; 4: +4; 5: +5 |
| 2 | Does your child currently participate in any extracurricular sports activities? | 1: Yes; 2: No | 1: +1; 2: +0 |
| 3 | What sport does your child practice (in case of extracurricular activity)? | Written responses | 0;1;2;3;4 **  No practice (0 METs): +0; 2–3.5 METs: + 1; 3.5–5 METs: +2; 5–6.5 METs: +3;>6.5 METs: + 4 |
| 4 | How many hours a week do you dedicate to practicing extracurricular activities? | Written responses | 0;1;2;3;4 **  No practice(0h): +0;<1.5h: +1; 1.5 –3h: +2; 3–4.5h: +3; >4.5h: +4 |
| 5 | During school hours, how many days per week does your child do physical activity? How long per day? | Written responses | 0;1;2;3;4**  No practice: +0;<1day x 1.5h: +1; 2days x 45min –75min: +2; 2days x 75 min– 2h: +3;>2days = +4 |
| 6 | Do you and your family participate in physical activities with your child?" | 1: Never; 2: Very sporadically; 3: In general, yes, but only a little;4: Often; 5: Almost every day; 6: Don't know or no comment. | 0;1;2;3;4 **  1 or 6: +0; 2: +1; 3: +2; 4: +3; 5: +4 |

* The answers are cumulative, contributing to an overall score that categorizes activity levels as follows: 2–9 points indicate a lightly active level;10–15 points indicate a moderately active level; and scores above 15 indicate very active level.

** Calculated according to METs (metabolic equivalents of task), defined as the ratio of the work metabolic rate to the resting metabolic rate (1 MET ≈ 3.5 mL O₂·kg⁻¹·min⁻¹)^3^_._

**Supplementary Table S2.** Biochemical parameters of subjects with the three phenotypes of PKU and control subjects ^1^

| Plasma biochemical parameters | cPKU  (n=22) | PKU-BH_4_  (n=21) | HPA  (n=19) | Controls  (n=20) | *p* |
| --- | --- | --- | --- | --- | --- |
| Iron profile test |  |  |  |  |  |
| Iron, μmol/L | 12.4 ± 4.9 | 14.2 ± 5.9 | 16.3 ± 6.1 | 11.3 ± 9.5 | 0.07 |
| Transferrin, mg/L | 2760 ± 693 | 2645 ± 681 | 2848 ± 327 | 1759 ± 1352 | 0.14 |
| Ferritin, μg/L | 43.2 ± 31.9 ^a^ | 42.4 ± 17.7 ^ab^ | 40.0 ± 17.8 ^ab^ | 23.6 ± 22.4 ^b^ | 0.030 |
| Plasma vitamins and minerals |  |  |  |  |  |
| Vitamin A, µmol/L | 1.5 ± 0.4 | 1.3 ± 0.7 | 1.2 ± 0.3 | 1.3 ± 0.3 | 0.23 |
| Vitamin D, ng/mL | 38.8 ± 13.9 ^a^ | 29.9 ± 13.2 ^ab^ | 26.9 ± 9.4 ^b^ | na | 0.001 |
| Vitamin E, µmol/L | 27.7 ± 8.7 | 24.6 ± 10.0 | 24.9 ± 10.5 | 26.0 ± 6.7 | 0.68 |
| Vitamin B_12_, pmol/L | 846 ± 258 ^a^ | 536 ± 298 ^b^ | 404 ± 128 ^b^ | 425 ± 134 ^b^ | <0.001 |
| Folic acid, nmol/L | 34.3 ± 7.9 ^a^ | 26.4 ± 11.2 ^b^ | 16.9 ± 6.8 ^c^ | 15.5 ± 6.6 ^c^ | <0.001 |
| Zinc, µg/L | 1103 ± 268 | 950 ± 287 | 939 ± 273 | 758 ± 418 | 0.17 |
| Selenium, µg/L | 85.4 ± 24.2 | 79.3 ± 15.1 | 81.9 ± 10.9 | 82.5 ± 9.1 | 0.78 |
| Polyunsaturated fatty acids^2^ |  |  |  |  |  |
| Linoleic acid, nmol/g Hb | 1574 ±340 | 1697 ±854 | 883±882 | 1017 ±835 | 0.18 |
| α-Linolenic acid, nmol/g Hb | 15 ±10 | 13 ±8 | 5 ± 5 | 7±7 | 0.11 |
| Arachidonic acid, nmol/g Hb | 2342± 400 ^a^ | 2328 ±942^ab^ | 1506 ±1488 ^b^ | 1765±1435 ^ab^ | 0.034 |
| Eicosapentaenoic acid, nmol/g Hb | 65 ±35 | 40 ±24 | 21 ±24 | 26±25 | 0.06 |
| Docosahexaenoic acid, nmol/g Hb | 915±336^a^ | 543±266^b^ | 300 ±309 ^abc^ | 344±299 ^c^ | 0.002 |
| Plasma amino acid profile |  |  |  |  |  |
| α-Alanine, µmol/L | 265 ±64 | 230 ±79 | 236±51 | 279±64 | 0.07 |
| L-carnitine, µmol/L | 33±13 ^ab^ | 31± 9 ^ab^ | 26 ±9^b^ | 35±7^a^ | 0.041 |
| Tryptophan, µmol/L | 56±14 | 53 ± 9 | 50± 7 | 59±14 | 0.09 |

^1^ All values are mean ± SD. Means in a row with superscripts without a common letter differ, *p*< 0.05 (Bonferroni post hoc test). ^2^ Composition of polyunsaturated fatty acids in erythrocytes. Hb, hemoglobin; IU, international units; na, not available.

**Supplementary Table S3.** Mediterranean Diet Quality Index according to PKU phenotype

|  | cPKU  (n=22) | PKU-BH_4_  Low-protein diet - LP  (n=10) | PKU-BH_4_  Normal diet - NP  (n= 11) | HPA  (n=19) | Controls  (n=20) | *p* |
| --- | --- | --- | --- | --- | --- | --- |
| KIDMED score | na | na | 2.55 ± 0.52 | 2.47 ± 0.51 | 2.60 ± 0.75 | 0.82 |
| KIDMED Score = 1 | na | na | 0 (0) | 0 (0) | 3 (15) | 0.014 |
| KIDMED Score =2 | na | na | 5 (45) ^a,b^ | 10 (53) ^a^ | 2 (10) ^b^ |  |
| KIDMED Score = 3 | na | na | 6 (55) | 9 (47) | 15 (75) |  |
| *Available KIDMED Questions for Individuals with PKU and Control Subjects* | | | | | | |
| Eats a fruit every day | 22 (100) | 10 (100) | 9 (82) | 16 (84) | 18 (90) | 0.23 |
| Has a second fruit every day | 15 (68) | 9 (82) | 5 (45) | 13 (68) | 14 (70) | 0.30 |
| Has fresh or cooked vegetables regularly once a day | 21 (95) | 10 (100) | 10 (91) | 17 (90) | 15 (75) | 0.18 |
| Has fresh or cooked vegetables more than once a day | 16 (73) | 8 (80) | 7 (64) | 11 (58) | 13 (65) | 0.75 |
| Uses olive oil at home | 22 (100) | 10 (100) | 11 (100) | 17 (90) | 20 (100) | 0.15 |
| Skips breakfast | 6 (27) | 1 (10) | 1 (9) | 3 (16) | 1 (5) | 0.31 |
| Has commercially baked goods or pastries for breakfast | 3 (14) | 1 (10) | 1 (9) | 3 (16) | 1 (5) | 0.85 |
| Eats sweets and candy several times every day | 8 (36) ^a^ | 2 (20) ^ab^ | 0 (0) ^bc^ | 1 (5) ^bc^ | 0 (0) ^c^ | 0.001 |
| Adapted KIDMED score (8 questions) | 4±1 | 4±1 | 4±1 | 3±1 | 4±1 | 0.50 |

^1^ All values are mean (SD) or n (%), as appropriate. Means in a row with superscripts without a common letter differ, *p* < 0.05. Data analyzed by one-factor ANOVA for continuous variables or chi-square test or Fisher test, as appropriate.

KIDMED, Mediterranean Diet Quality Index in children and adolescents. KIDMED score = 1 corresponds to a score of ≤3, indicating very low-quality diet quality; KIDMED score = 2 corresponds to a score of 4–7, indicating “improvement needed to adjust intake to Mediterranean patterns”; KIDMED score = 3 corresponds to a score of ≥8, indicating an optimal Mediterranean diet.

**Supplementary Table S4.** Pearson’s correlations of carbohydrate, total fat and SFA intake with biochemical parameters, stratified by PKU phenotype and control group.

|  | **cPKU**  **(n=22)** | **PKU-BH_4_**  **(n=21)** | **HPA**  **(n=19)** | **Control group**  **(n=20)** |
| --- | --- | --- | --- | --- |
| **Carbohydrate consumption** | | | | |
| Total Cholesterol, mg/dl | -0.04 | -0.38 | 0.16 | 0.02 |
| LDL-C, mg/dL | -0.02 | -0.28 | 0.04 | -0.18 |
| HDL-C, mg/dL | -0.05 | -0.42† | 0.22 | 0.32 |
| Non-HDL-C, mg/dL | 0.07 | -0.18 | 0.07 | -0.18 |
| Triglycerides, mg/dL | 0.13 | 0.14 | 0.20 | 0.02 |
| Triglyceride/HDL-C ratio | 0.11 | 0.28 | 0.04 | -0.14 |
| Triglyceride-glucose index | 0.04 | 0.13 | 0.17 | 0.03 |
| Glucose, mg/dL | -0.33 | 0.03 | -0.12 | 0.03 |
| Insulin, mU/L | 0.08 | 0.24 | 0.46† | -0.06 |
| HOMA-IR | 0.03 | 0.23 | 0.44† | -0.06 |
| **Total fat consumption** | | | | |
| Total Cholesterol, mg/dl | 0.40† | 0.39† | 0.44† | -0.12 |
| LDL-C, mg/dL | 0.43* | 0.34 | 0.37 | -0.05 |
| HDL-C, mg/dL | 0.61* | 0.29 | 0.27 | -0.18 |
| Non-HDL-C, mg/dL | 0.21 | 0.27 | 0.41 | -0.03 |
| Triglycerides, mg/dL | -0.06 | -0.04 | 0.21 | 0.04 |
| Triglyceride/HDL-C ratio | -0.33 | -0.14 | 0.03 | 0.12 |
| Triglyceride-glucose index | 0.02 | -0.08 | 0.14 | 0.03 |
| Glucose, mg/dL | 0.26 | -0.24 | -0.32 | 0.03 |
| Insulin, mU/L | 0.27 | -0.48* | 0.30 | 0.22 |
| HOMA-IR | 0.30 | -0.47* | 0.27 | 0.21 |
| **SFA consumption** | | | | |
| Total Cholesterol, mg/dl | 0.10 | 0.56* | -0.15 | 0.15 |
| LDL-C, mg/dL | 0.03 | 0.54* | -0.09 | 0.24 |
| HDL-C, mg/dL | 0.09 | 0.15 | -0.20 | -0.14 |
| Non-HDL-C, mg/dL | 0.08 | 0.53* | -0.07 | 0.27 |
| Triglycerides, mg/dL | -0.05 | 0.36 | 0.07 | 0.21 |
| Triglyceride/HDL-C ratio | -0.07 | 0.20 | 0.16 | 0.22 |
| Triglyceride-glucose index | 0.003 | 0.41† | 0.11 | 0.19 |
| Glucose, mg/dL | 0.19 | -0.19 | 0.14 | 0.009 |
| Insuline, mU/L | 0.42* | -0.15 | -0.14 | 0.24 |
| HOMA-IR | 0.44* | -0.16 | -0.12 | 0.23 |

* *p*< 0.05; ** *p*< 0.001; † *p* = 0.05–0.09.

HDL-C, high-density lipoprotein cholesterol; HOMA-IR, homeostatic model assessment insulin resistance; LDL-C, low-density lipoprotein cholesterol; PKU, phenylketonuria; SFA, saturated fatty acids.

**Supplementary Table S5.** Anthropometric and plasma biochemical parameters stratified by dietary treatment in PKU participants with and without low-protein diet treatment and the control group ^1^

|  | PKU  Normal diet  (n=30) | PKU  Low-protein diet  (n=32) | Control group  (n=20) | *p* |
| --- | --- | --- | --- | --- |
| Age, years | 9.3±4.0 | 10.5±4.3 | 10.3±3.8 | 0.52 |
| Sex, female (%) | 19(63) | 18(56) | 9(45.0) | 0.44 |
| Anthropometric parameters | | | | |
| Weight Z-score | 0.2±1.4 | 0.1±1.1 | 0.4±1.2 | 0.72 |
| Height Z-score | -0.1±1.2 | -0.2±1.1 | -0.1±0.9 | 0.84 |
| BMI Z-score | 0.2±1.3 | 0.4±1.1 | 0.6±1.5 | 0.56 |
| Plasma lipid profile, glycemic markers, and insulin resistance parameters | | | | |
| Total Cholesterol, mg/dL | 158±24^a^ | 141 ±26 ^b^ | 156 ± 20 ^ab^ | 0.009 |
| LDL-C, mg/dL | 87±18^a^ | 75 ±20 ^b^ | 91±16^ab^ | 0.004 |
| HDL-C, mg/dL | 60±12 ^a^ | 52±11 ^b^ | 54 ±10^ab^ | 0.013 |
| Non-HDL-C, mg/dL | 98 ±20^ab^ | 88±22^b^ | 102±17^a^ | 0.038 |
| Triglycerides, mg/dL | 57 ±25 | 60 ±22 | 55±17 | 0.67 |
| Triglyceride/HDL-C ratio | 1.0±0.6 | 1.2±0.5 | 1.1±0.4 | 0.38 |
| Triglyceride-glucose index | 4.2±0.2 | 4.2±0.2 | 4.2±0.1 | 0.72 |
| Glucose, mg/dL | 85± 7 | 86±10 | 87± 7 | 0.60 |
| Insulin, mU/L | 4.3±4.1 | 5.0±3.5 | 5.2±5.0 | 0.65 |
| HOMA-IR | 0.9±0.8 | 1.1±0.8 | 1.2±1.3 | 0.61 |
| Plasma vitamins and minerals | | | | |
| Vitamin A, µmol/L | 1.1±0.4^b^ | 1.5±0.5^a^ | 1.3±0.3^ab^ | 0.030 |
| Vitamin B_12_, pmol/L | 383 ±122 ^b^ | 815 ±273 ^a^ | 425±134^b^ | <0.001 |
| Vitamin D, ng/mL | 26.3±9.8^b^ | 37.6±13.9^a^ | na | <0.001 |
| Vitamin E, µmol/L | 25.3±11.1 | 26.3±8.2 | 26.0±6.7 | 0.90 |
| Folic acid, nmol/L | 17.3±7.4^b^ | 34.8±6.7^a^ | 15.5±6.6^b^ | <0.001 |
| Zinc, µg/L | 961 ±243 | 1038±314 | 758 ±418 | 0.17 |
| Selenium, µg/L | 82.6±11.6 | 81.9±22.3 | 82.5±9.1 | 0.74 |
| Polyunsaturated fatty acids^2^ | | | | |
| Linoleic acid, nmol/g Hb | 1164±888 | 1628±632 | 1017 ±835 | 0.37 |
| α-Linolenic acid, nmol/g Hb | 7 ± 6 ^b^ | 15 ±10^a^ | 7±7^ab^ | 0.037 |
| Arachidonic acid, nmol/g Hb | 1872 ±1386 ^b^ | 2277 ±618 ^a^ | 1765±1435 ^ab^ | 0.002 |
| Eicosapentaenoic acid, nmol/g Hb | 29 ±25 | 56±35 | 26±25 | 0.23 |
| Docosahexaenoic acid, nmol/g Hb | 380 ±293 ^ab^ | 808 ±366^a^ | 344±299 ^b^ | 0.007 |
| Iron profile test |  |  |  |  |
| Iron, μmol/L | 15.8±6.5^a^ | 12.8±4.5^ab^ | 11.3±9.5^b^ | 0.024 |
| Transferrin, mg/L | 2849±308 | 2653±770 | 1759±938 | 0.06 |
| Ferritin, μg/l | 40.0±18.1^a^ | 43.8±27.7^a^ | 23.6±22.4^b^ | 0.01 |
| Plasma amino acid profile |  |  |  |  |
| Phenylalanine, µmol/L | 231±93 ^b^ | 405 ±225^a^ | 58.9±12.3 ^c^ | <0.001 |
| Tyrosine, µmol/L | 58 ±11^b^ | 45±15^c^ | 67 ±13 ^a^ | <0.001 |
| Alanine, µmol/L | 238 ±51 | 250±79 | 279±64 | 0.054 |
| L-carnitine, µmol/L | 237±9 ^b^ | 250±79^ab^ | 279±64 ^a^ | 0.016 |
| Tryptophan, µmol/L | 52 ± 8 | 54±13 | 59±14 | 0.12 |
| Phe/Tyr ratio | 4.2±2.1 ^b^ | 9.8±6.2 ^a^ | 0.9±0.1 ^c^ | <0.001 |

^1^ All values are mean ± SD or n (%), as appropriate. Means in a row with superscripts without a common letter differ, *p*< 0.05 (Bonferroni post hoc test). ^2^ Composition of polyunsaturated fatty acids in erythrocytes. IU, international units; na, not available.

**Supplementary Table S6**. Energy and macronutrient intake stratified by dietary treatment in subjects with PKU and the control group ^1^

|  | PKU  Normal diet  (n=30) | PKU  Low protein diet  (n=32) | Control group  (n=20) | *p* |
| --- | --- | --- | --- | --- |
| Energy, kcal/day | 1678 (1463–1931) | 1798 (1566–2209) | 1755 (1491–1842) | 0.47 |
| Total protein, % | 14.3 (12.5–15.9) | 15.1 (11.5–18.7) | 15.0 (13.3–16.8) | 0.64 |
| Natural protein, % | 14.3 (12.5–15.5) ^a^ | 3.0 (2.2–4.4) ^b^ | 15.0 (13.3–16.8) ^a^ | <0.001 |
| Phenylalanine, mg/day | 2197 (1412 – 2666) ^a^ | 506 (380 – 698) ^b^ | 1615 (1369 – 1991) ^a^ | <0.001 |
| Tyrosine, mg/day | 796 (682 – 1277) ^b^ | 5486 (3560 – 7225) ^a^ | 712 (605 – 841) ^b^ | <0.001 |
| Carbohydrates, % | 46.2 (42.4–49.4) ^b^ | 54.9 (50.5–57.7) ^a^ | 47.5 (44.2–50.7) ^b^ | <0.001 |
| Sugars, % | 10.0 (8.6–12.7) ^c^ | 17.7 (14.3–21.2) ^a^ | 13.7 (11.6–18.3) ^b^ | <0.001 |
| Fiber, g/day | 12.8 (10.7–17.0) ^b^ | 22.4 (16.1–28.9) ^a^ | 14.3 (10.4–16.9) ^b^ | <0.001 |
| Starch, g/day ^2^ | 24.1 (17.5–40.4) | 19.9 (12.2–32.0) | 15.6 (8.7–25.3) | 0.12 |
| Total fat, % | 39.4 (34.7–41.0) ^a^ | 32.2 (26.7–36.8) ^b^ | 37.5 (33.1–40.2) ^a^ | <0.001 |
| SFAs, % | 11.2 (9.7–12.4) | 9.3 (7.3–14.5) | 11.3 (9.0–12.6) | 0.49 |
| MUFAs, % | 14.6 (13.0–17.9) ^a^ | 12.7 (9.8–16.5) ^ab^ | 11.6 (10.4–12.8) ^b^ | <0.001 |
| PUFAs, % | 3.9 (3.2–4.5) | 3.5 (2.8–4.7) | 3.9 (3.0–4.4) | 0.86 |
| Cholesterol, mg/day ^3^ | 215 (144–297) ^a^ | 13 (6–32) ^b^ | 164 (128–208) ^a^ | <0.001 |

^1^ All values are medians (IQR). Medians in a row with superscripts without a common letter differ, p < 0.05. Data analyzed by Kruskal–Wallis and Mann–Whitney post hoc test. ^2^ Data on starch is not available in SLPFs. ^3^ Data on cholesterol is not available in SLPFs and PFAAs. MUFAs, monounsaturated fatty acids; PFAAs, precursor-free L-amino acid supplement; PUFAs, polyunsaturated fatty acids; SFAs, saturated fatty acids; SLPFs, special low-protein foods.

**Supplementary Table S7.** Multivariate linear regression models for association between plasma biochemical parameters and natural protein intake (%).

| **Natural protein, %** | **β (95% CI)** |
| --- | --- |
| **Plasma lipid profile, glycemic markers, and insulin resistance parameters** | |
| Total Cholesterol, mg/dl | 1.09 (0.13–2.05)* |
| LDL-C, mg/dL | 0.95 (0.23–1.67)* |
| HDL-C, mg/dL | 0.32 (-0.14–0.79) |
| Non-HDL-C, mg/dL | 0.77 (-0.02–1.56) |
| Triglycerides, mg/dL | -0.40 (-1.29–0.48) |
| Triglyceride/HDL-C ratio | 0.00 (-0.01–0.00) |
| Triglyceride-glucose index | -0.01 (-0.03–0.01) |
| Glucose, mg/dL | 0.02 (-0.34–0.37) |
| Insulin, mU/L | -0.04 (-0.20–0.12) |
| HOMA-IR | 0.00 (-0.04–0.04) |
| **Plasma vitamins and minerals** | |
| Vitamin B_12_, pmol/L | -34.19 (-42.98–-25.40)*** |
| Vitamin A, µmol/L | -0.01 (-0.03–0.01) |
| Vitamin D, ng/mL | -1.68 (-2.30–-1.06)*** |
| Vitamin E, µmol/L | 0.00 (-0.39–0.39) |
| Folic Acid, nmol/L | -1.38 (-1.69–-1.07)*** |
| Zinc, µg/L | -16.26 (-29.66–-2.86)* |
| Selenium, µg/L | 0.46 (-0.20–1.12) |
| **Polyunsaturated fatty acids** | |
| Linoleic acid, nmol/g Hb | -42.11 (-75.86–-8.36)* |
| α-Linolenic acid, nmol/g Hb | -0.72 (-1.06–-0.38)*** |
| Arachidonic acid, nmol/g Hb | -39.88 (-89.49–9.73) |
| Docosahexaenoic acid, nmol/g Hb | -39.96 (-53.55–-26.36)*** |
| Eicosapentaenoic acid, nmol/g Hb | -2.66 (-3.89–-1.44)*** |
| **Iron profile** |  |
| Iron, μmol/l | 0.02(-0.29-0.32) |
| Ferritin, μg/l | -0.77 (-1.78–0.25) |
| Transferrin, mg/L | -18.98 (-59.06–21.10) |
| **Plasma amino acid profile** | |
| Alanine, µmol/L | 1.26 (-1.58–4.10) |
| L-carnitine, µmol/L | -0.32 (-0.73–0.10) |
| Phenylalanine, µmol/L | -19.89 (-27.04–-12.74)*** |
| Tyrosine, µmol/L | 1.59 (1.01–2.17)*** |
| Tryptophan, µmol/L | 0.03 (-0.46–0.52) |
| Ratio Phe/Tyr | -0.60 (-0.79–-0.41)*** |

Models are adjusted for sex, age (years), weight (kg), height (cm), physical activity, and macronutrients intake (%). HDL-C, high-density lipoprotein cholesterol; LDL-C, low-density lipoprotein cholesterol; non-HDL-C, non-high-density lipoprotein cholesterol. *p < 0.05, **p < 0.01, ***p < 0.001.

**REFERENCES**

1. van Wegberg AMJ, MacDonald A, Ahring K, et al. European guidelines on diagnosis and treatment of phenylketonuria: First revision. *Mol Genet Metab*. 2025;145(2):109125. doi:10.1016/J.YMGME.2025.109125

2. Jurado-Castro JM, Llorente-Cantarero FJ, Gil-Campos M. Evaluación de la actividad física en niños. *Acta Pediatr Esp*. 2019;77(5/6):94-99.

3. Butte NF, Watson KB, Ridley, et al. A Youth Compendium of Physical Activities: Activity Codes and Metabolic Intensities. *Med Sci Sports Exerc*. 2018;50(2):246-256. doi:10.1249/MSS.0000000000001430
